# Supplementary material for: Antifungal susceptibility of Candida species to copper oxide nanoparticles on polycaprolactone fibers (PCL-CuONPs)
Source: PLoS One. 2020 Feb 24;15(2):e0228864. doi: 10.1371/journal.pone.0228864 (PMC7039411; doi:10.1371/journal.pone.0228864)
Supplement: Attachment — Submitted filename: Response comments 19-oct-2019.docx [file pone.0228864.s001.docx]

**Response comments**

1. Is the manuscript technically sound, and do the data support the conclusions?

The manuscript must describe a technically sound piece of scientific research with data that supports the conclusions. Experiments must have been conducted rigorously, with appropriate controls, replication, and sample sizes. The conclusions must be drawn appropriately based on the data presented.

Reviewer #1: Partly

Response: The corrections were made in the manuscript, figures and discussion were attached, the summary as well as the conclusions were modified

2. Has the statistical analysis been performed appropriately and rigorously?

Reviewer #1: N/A

3. Have the authors made all data underlying the findings in their manuscript fully available?

The [PLOS Data policy](http://www.plosone.org/static/policies.action#sharing) requires authors to make all data underlying the findings described in their manuscript fully available without restriction, with rare exception (please refer to the Data Availability Statement in the manuscript PDF file). The data should be provided as part of the manuscript or its supporting information, or deposited to a public repository. For example, in addition to summary statistics, the data points behind means, medians and variance measures should be available. If there are restrictions on publicly sharing data—e.g. participant privacy or use of data from a third party—those must be specified.

Reviewer #1: No

Response: the corrections were made in the manuscript,

4. Is the manuscript presented in an intelligible fashion and written in standard English?

PLOS ONE does not copyedit accepted manuscripts, so the language in submitted articles must be clear, correct, and unambiguous. Any typographical or grammatical errors should be corrected at revision, so please note any specific errors here.

Reviewer #1: No

Response: the corrections were made in the manuscript

5. Review Comments to the Author

Please use the space provided to explain your answers to the questions above. You may also include additional comments for the author, including concerns about dual publication, research ethics, or publication ethics. (Please upload your review as an attachment if it exceeds 20,000 characters)

Reviewer #1: In general, the manuscript was written very poorly. Likewise, explanations of the results are rather shallow or not explained at all.
1. Some phrases in the conclusion is not supported in the results or vague
(a) “…diameter were affected by the increment of CuONPs concentration.” This is not supported by the data You only show with and without CuONP, no “increment” or varying CuONP concentrations was presented.
(b) “….their cellular morphology was demonstrated to be affected by PCL-CuONPs composites determining a dose-dependent activity”. Please rephrase, the sentence construction is vague.
2. I have not seen the raw data of the UV-VIS spectra . (No attached supplementary data.)
3. The use of English language needs improvement. Many sentences are vague. You need the help of a professional grammar editor.
4. The SEM images do not support that the fibers are nanometer in thickness. Although there is no “nanofiber” word in the title, the word “nanofiber” was used to describe the fiber samples in some parts of the manuscript (e.g., abstract, results and discussion, conclusion).
5. Title: You can remove the word “oral” in the title, “Candida Species” is enough.
6. Abstract: Line 13: “Polycaprolactone-silver…”. Silver was written instead of Copper.
7. Abstract: Line 16-17: “the second step involved the simple addition of polycaprolactone before electrospinning process”. The sentence is vague. Please rephrase. I think you mean that the second step involved electrospinning using polycaprolactone maybe as matrix of the CuO.
8. Lines 82-86 (Methodology: Copper oxide nanoparticle preparation): Some phrases sentences are vague and confusing. Simplify the sentence construction.
9. The caption of Figure 1 is poorly written. The figure labels (a,b,c) should be written also in the “caption of the figure” to concretely match its label/description.
10. Figure 1a is not an UV-VIS spectrum but rather the intensity of a certain peak from the UV-VIS spectrum. Rewrite the description more concretely.
11. Figure 1a: Is the x-axis really CuONP concentration? Or it is the initial copper nitrate concentration used?
12. Figure 1a: Does the label “270”, “275”, “280”, “285” pertains to the peak absorbance or wavelength position of the peak? The unit of your y-axis is “absorbance” while in the manuscript the unit is nm.
13. The explanation of Figure 1a in the manuscript is very shallow. What possible cause the shift? Instead of just saying “The observe changes in the spectrum reflects the characteristic pattern of CuONP formation…”.
14. Figure 1b is not merely the “size” but rather the frequency distribution of the samples diameter as measured by DLS.
15. The concentration labels in Figure 1b are not specified that is the initial concentration of copper nitrate.
16. “Figure 1a” and “Figure 1b” are not “called/mentioned” in the body of the manuscript
17. What is the purpose of having Raman Spectrum in your paper? You have not detected presence of CuO using Raman Spectroscopy. Likewise, you have already used commercially available PCL. Do you need to still identify that you have PCL using Raman Spectroscopy? Or do you need to identify the crystalline of your sample? Is determining the crystallinity relevant to your study?
18. Line 159-162: PCL is semicrystalline polyester. However, are you sure that what you have mentioned to be “narrow” peaks really are narrow to be able to signify “crystallinity” of PCL? Do you need to specify the crystalline of your sample in your paper?
19. Line 162. “The broad peak at 825 cm-1 indicates that the amorphous phase is present in the PCL fibers (Baranowska).” Where is the broad peak at 825 cm-1 (Figure 1c)? Do you forget to label or it is not present ?
20. Based from Figure 2b, the average diameter of the synthesized copper particles decreases with the increase in initial copper nitrate concentration. Briefly explain the possible reason of this trend.
21. Your Figure 2 has labels “a” and “b” but the figure caption does not have corresponding labels.
22. Line 165: “….free area of beads, precipitates and fractures.” Rephrase this phrase, I think you mean “with no beads, precipitates and fractures”.
23. Line 167-169: “Results obtained by electron microscopy showed the existing relation between size distribution and concentration of CuO nanoparticles.” This statement has no supporting explanations.
24. Figure 3: “PCL-CuO (mM) 25mM, 50mM….” The “mM” pertains to the initial copper nitrate concentration used in fabricating CuO, and not the concentration of CuO in PCL fiber? If so, the label “PCL-CuO (mM) might be misleading”.
25. What do you mean by the “inverted bracket” in Figure 3? Please specify in its caption.
26. The captions of Figures 4,5 and 6 are vague. They do not give specifications of what is a, b, c, and d”. Please rewrite the captions.
27. Figures 4,5, and 6 were described in the manuscript without identifier of which image (a,b,c or d) they are taking about. For example, they should have state that Figure 4a (instead of just Figure 4) is the SEM image of C.Albican which was exposed to polymer fiber without the CuO.
28. Line 194: “The SEM images of C. glabrata, as well as C. albicans, show the integrated cell wall of the yeast in the control conditions in Fig. 5 ” No need to write “as well as C. albican” since it is not part of Figure 5. If you want to write “as well as C. Albicans”, rephrase the sentence.

Response:  the corrections were made in the manuscript

6. PLOS authors have the option to publish the peer review history of their article ([what does this mean?](https://journals.plos.org/plosone/s/editorial-and-peer-review-process#loc-peer-review-history)). If published, this will include your full peer review and any attached files.


If you choose “no”, your identity will remain anonymous but your review may still be made public.


**Do you want your identity to be public for this peer review?** For information about this choice, including consent withdrawal, please see our [Privacy Policy](https://www.plos.org/privacy-policy).

Reviewer #1: No
